# Supplementary material for: From spots to cells: Cell segmentation in spatial transcriptomics with BOMS
Source: PLoS One. 2025 Jun 12;20(6):e0311458. doi: 10.1371/journal.pone.0311458 (PMC12161567; doi:10.1371/journal.pone.0311458)
Supplement: S1 Appendix — (PDF) [file pone.0311458.s001.pdf]

Supporting Information

Reproducibility Analysis of BOMS with Parameter Variations

Figures S1 and S2 demonstrate the effect of varying the tunable parameters of BOMS (K, h_s, and h_r) on the segmentation results for the Allen smFISH and osmFISH datasets, respectively. Subplot A in each figure presents heatmaps of normalized mutual information (NMI) with respect to the Silver Standard across different parameter combinations. Each heatmap corresponds to a specific spatial bandwidth (h_s), with rows and columns representing the range bandwidth (h_r) and the number of neighbors (K). The midpoint of the heatmap scale is set to the NMI obtained using the reference settings ($K = 30$, $h_s = 17.5$, $h_r = 0.4$ for Allen smFISH, and $K = 30$, $h_s = 6.5$, $h_r = 0.2$ for osmFISH).

Subplots B, C, and D in both figures show visual segmentation results for the reference settings and two additional parameter combinations. Despite the variation in parameters, the visual results remain highly similar, underscoring the robustness of BOMS to parameter changes. Notably, the spatial bandwidth (h_s) appears to have the most significant effect on segmentation outcomes, while the range bandwidth (h_r) and number of neighbors (K) have more subtle influences. These results demonstrate that BOMS achieves reproducible segmentation across a range of parameter settings.

Runtime Performance of BOMS on Large Datasets

To evaluate the runtime scalability of BOMS, we used the Allen smFISH dataset (22 genes) and cropped it to include 1 million points. This cropped section was appended to itself in the xy-space to create progressively larger datasets containing up to 20 million points. BOMS was then run on these datasets on two systems:

- **Dell XPS Laptop**– Specifications: Intel(R) Core(TM) i7 CPU @ 2.60GHz, 32 GB RAM. Due to memory limitations, runs were conducted on datasets up to 5 million points.
- **Workstation**– Specifications: Intel(R) Xeon(R) Gold 6254 CPU @ 3.10GHz, 541 GB RAM. Runs were conducted on datasets ranging from 1 million to 20 million points.

The runtime results are presented in Figure S3, showing an approximately linear increase in processing time with dataset size on the workstation system. For example, processing 1 million points took approximately 31.7 seconds, while processing 20 million points required 1066.3 seconds. Minor deviations from linearity are observed, likely due to system-level factors such as memory management and computational overhead. The laptop system showed similar trends but was limited by memory availability during the single-linkage clustering step, which uses scipy.cluster.hierarchy.fclusterdata.

These results demonstrate that BOMS scales approximately linearly with dataset size on appropriately equipped systems, highlighting its potential for efficient processing of large spatial transcriptomics datasets.

Challenging Cases and Limitations of BOMS

This figure highlights specific challenges faced by BOMS in segmenting cells under two extreme scenarios: (1) transcriptionally homogeneous regions, where adjacent cells with similar molecular profiles are undersegmented into a single cell; and (2) regions with high intracellular transcriptional heterogeneity, where BOMS oversegments a single cell into multiple smaller segments. These challenges are visualized using the NGE-based PCA coloring, where similar transcriptional signatures are represented by similar colors. While these cases underscore the inherent limitations of BOMS, such edge cases are expected and represent a broader challenge for segmentation algorithms in spatial transcriptomics.

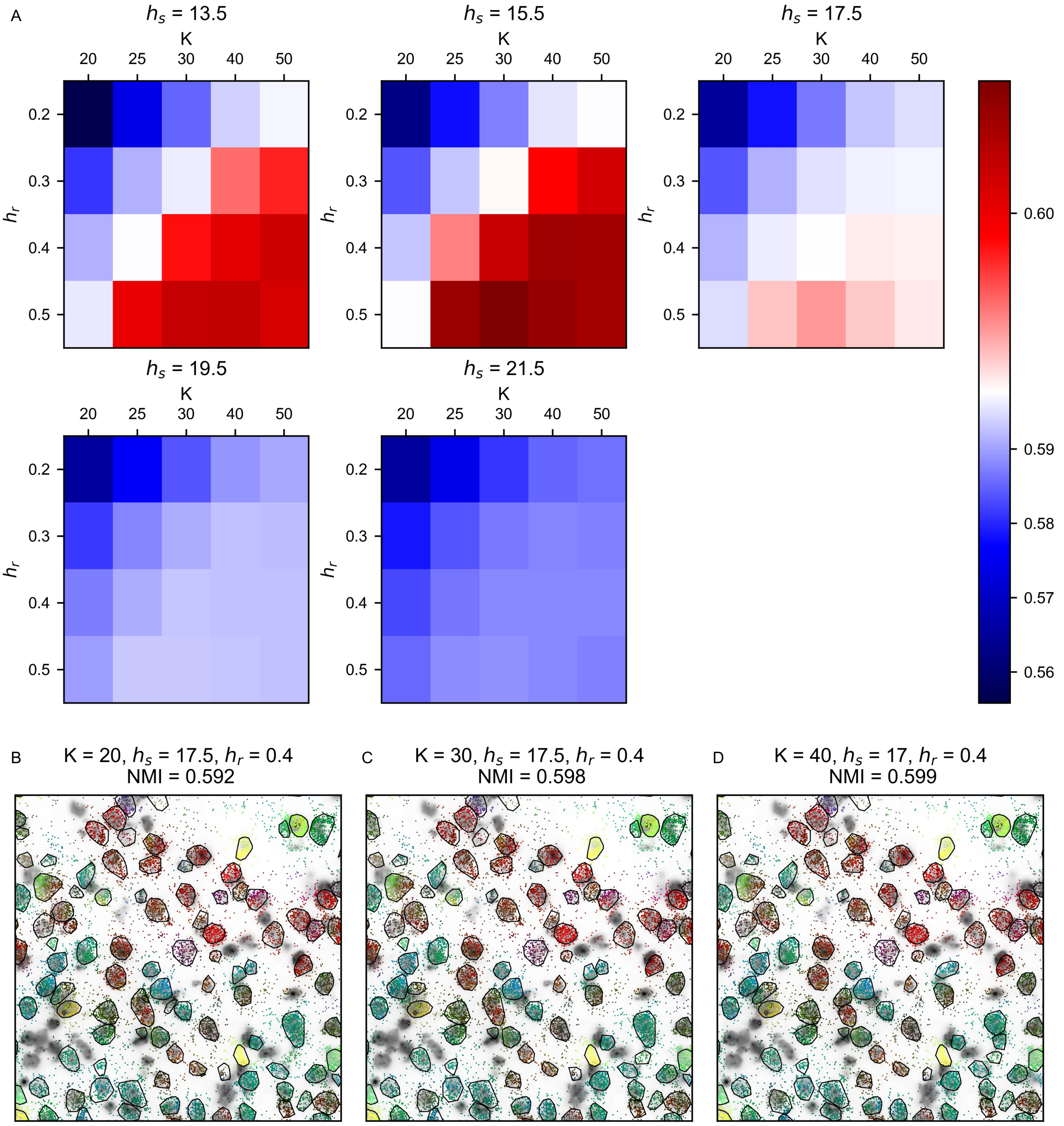

Figure S1.Impact of Parameter Variations on BOMS Results for Allen smFISH. A: Heatmaps of normalized mutual information (NMI) with respect to the Silver Standard for different parameter combinations. Each subplot corresponds to a specific spatial bandwidth (h_s), with rows and columns representing the range bandwidth (h_r) and the number of neighbors (K), respectively. The NMI for the reference settings ($K = 30$, $h_s = 17.5$, $h_r = 0.4$) is used as the midpoint of the heatmap scale. B, C, and D: Visual results of cell segmentation obtained using three different parameter combinations, highlighting the similarity in results despite parameter variations.

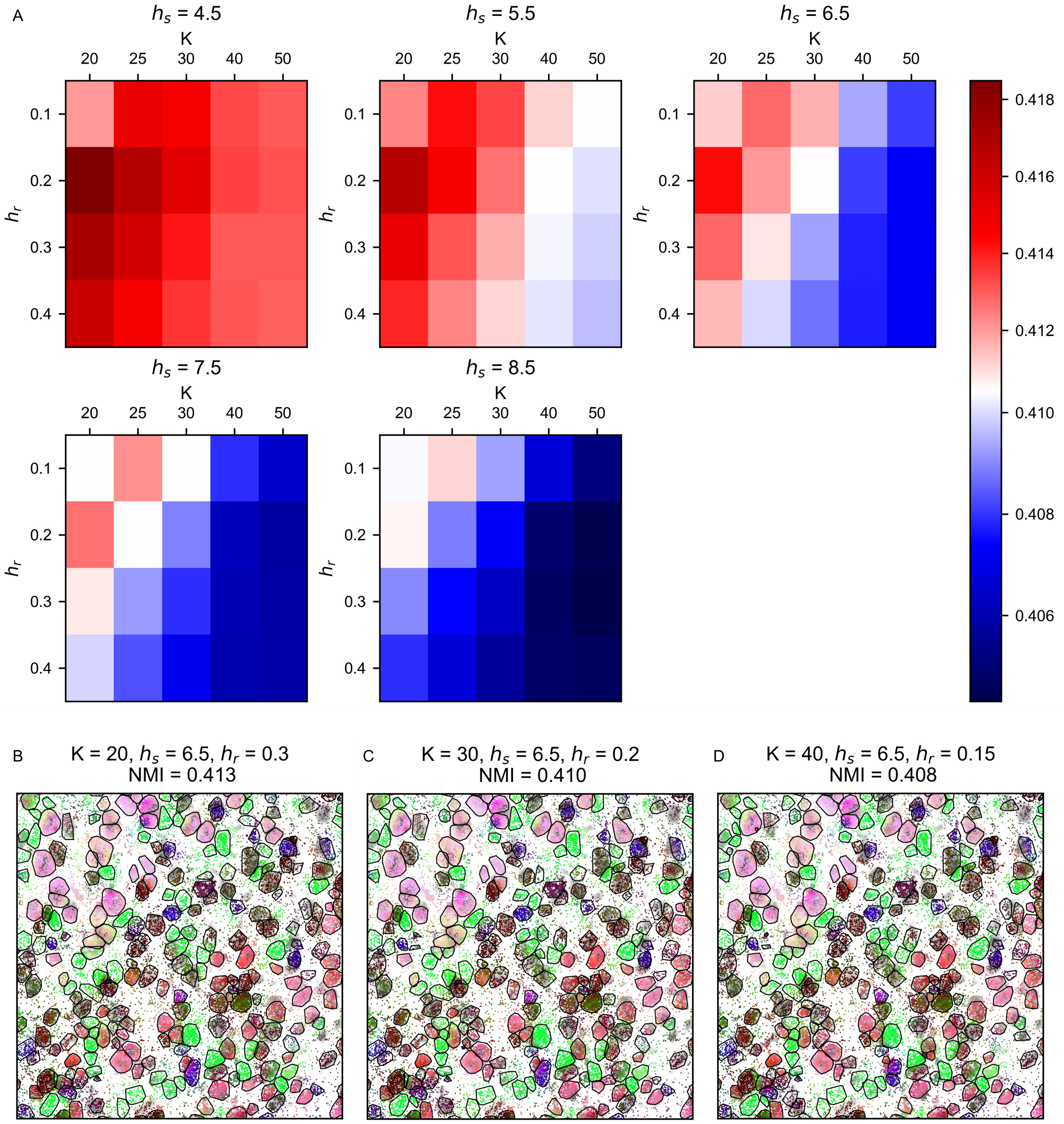

Figure S2. Impact of Parameter Variations on BOMS Results for osmFISH. A: Heatmaps of normalized mutual information (NMI) with respect to the Silver Standard for different parameter combinations. Each subplot corresponds to a specific spatial bandwidth (h_s), with rows and columns representing the range bandwidth (h_r) and the number of neighbors (K), respectively. The NMI for the reference settings ($K = 30$, $h_s = 6.5$, $h_r = 0.2$) is used as the midpoint of the heatmap scale. B, C, and D: Visual results of cell segmentation obtained using three different parameter combinations, emphasizing the robustness of BOMS to parameter variations.

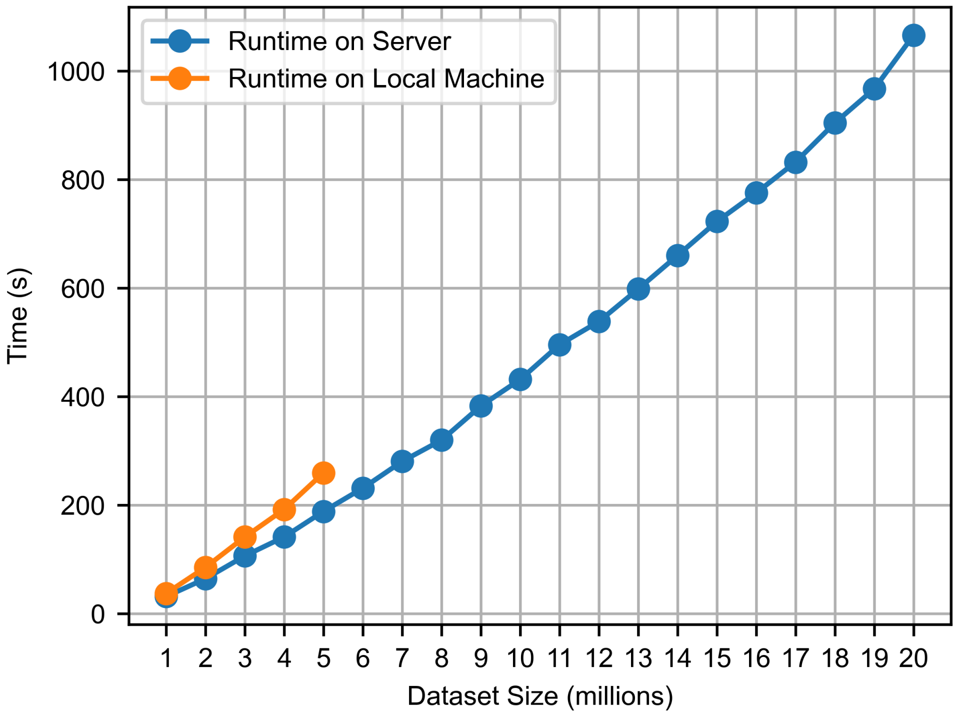

Figure S3. Runtime Performance of BOMS. The figure shows the runtime of BOMS on progressively larger datasets, evaluated on a workstation (up to 20 million points) and a Dell XPS laptop (up to 5 million points). Runtime scales approximately linearly with dataset size, with minor deviations due to system factors. The workstation demonstrates the capability to handle significantly larger datasets, while the laptop system is limited by memory constraints during the single-linkage clustering step.

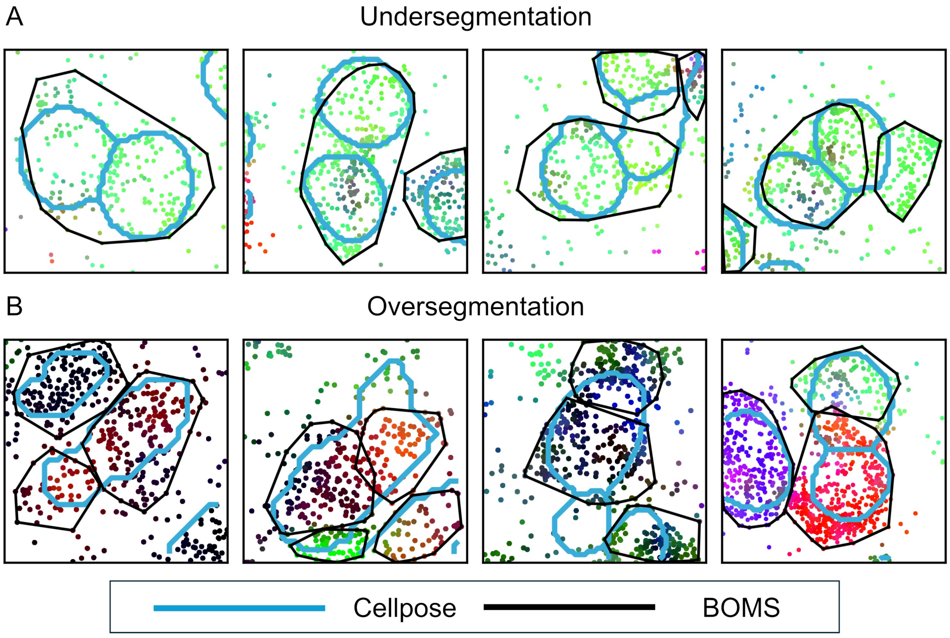

Figure S4. Challenging cases for BOMS. BOMS performance can be impacted in transcriptionally homogeneous regions or regions with intracellular transcriptional heterogeneity. Molecules are visualized by coloring spots based on a PCA projection of the NGE vectors, highlighting transcriptional similarity or dissimilarity. Black contours represent cell boundaries obtained from BOMS, while cyan contours represent boundaries obtained from Cellpose. A: Examples of undersegmentation where adjacent cells with similar transcriptional signatures are merged into a single cell. B: Examples of oversegmentation where intracellular transcriptional heterogeneity causes a single cell to be split into multiple segments.

Cell Type Label Transfer from Silver Standard - MERFISH Dataset

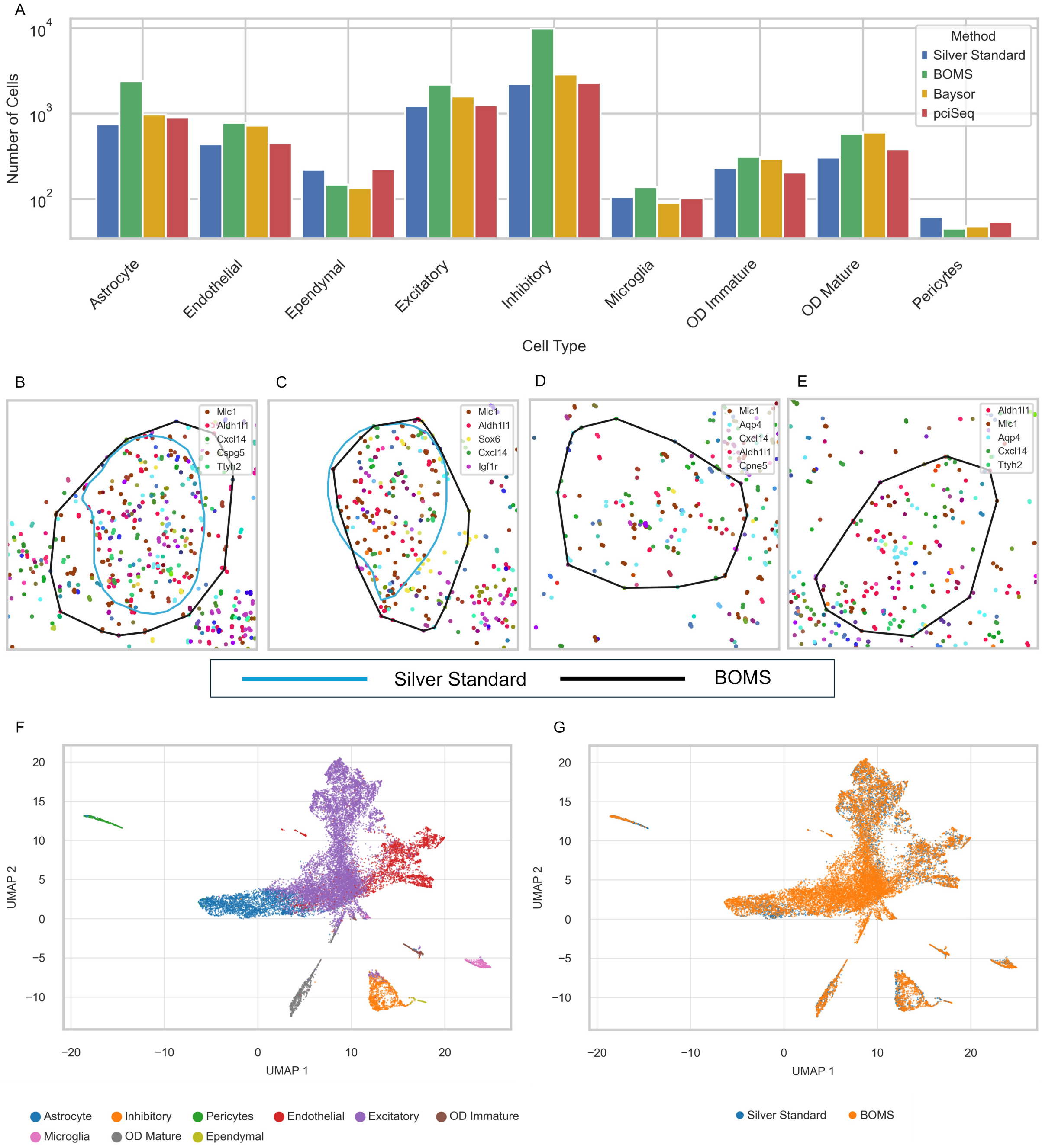

Figure S5. Cell type label transfer for the MERFISH dataset. A: Bar plot showing the number of cells assigned to each cell type by the Silver Standard, BOMS, Baysor, and pciSeq. B-C: Examples of astrocyte cells identified by both the Silver Standard and BOMS. D-E: Examples of astrocytes detected exclusively by BOMS. F-G: Joint UMAP embedding of cells from BOMS and the Silver Standard colored by (F) cell types and (G) dataset, demonstrating additional recovery by BOMS.

Co-occurrence of marker genes from different lineages

As an independent assessment of segmentation quality, we evaluated the extent to which cells erroneously co-express marker genes that are normally restricted to distinct lineages. Because a correctly segmented cell should belong to a single lineage, cells that simultaneously express markers from different lineages indicate misassignment of transcripts. We focus on four illustrative gene pairs:

- **Hexb and Slc32a1**: Hexb is a specific marker for Microglia (glial lineage), whereas Slc32a1 is expressed in inhibitory neurons. Since glial and neuronal cells are distinct, co-expression of these markers should be minimal.
- **Pdgfra and Gfap**: Pdgfra marks Oligodendrocyte Precursor Cells, while Gfap is a marker for Astrocytes. Although both are glial, they represent separate lineages and are not expected to be co-expressed in the same cell.
- **Ttr and Gad2**: Ttr is predominantly expressed in choroid plexus (epithelial) cells, whereas Gad2 is a marker for inhibitory neurons. Epithelial and neuronal cells should have non-overlapping marker expression.
- **Ttr and Pdgfra**: Here, Ttr (choroid plexus epithelium) is contrasted with Pdgfra (oligodendrocyte precursor cells), reflecting the expected separation between epithelial and glial cells.

Methodology:
To quantify marker co-occurrence, we generated scatter plots of gene expression for each pair across segmented cells. The following steps were used to determine expression thresholds and compute contamination metrics:

1. **Threshold Determination:** For each gene, we identified a reference set of cells corresponding to its expected cell type (e.g., cells annotated as Microglia for Hexb) in the Silver Standard. We then examined the distribution of expression values in these "true" cells and set the threshold at the 20th percentile. This choice ensures that approximately 80% of the true cells express the marker above the threshold, while only a small fraction of cells from other lineages do.
2. **Scatter Plot Analysis:** For each gene pair, cells are plotted based on their expression (counts) of the two markers. The thresholds are overlaid as vertical/horizontal dashed lines.
3. **Contamination Metrics:** We record the number of cells (n) that exceed the threshold for both genes. Additionally, we define a contamination score s as: $d = \frac{n_{double}}{n_{only\ gene\ 1} + n_{only\ gene\ 2}}$, where n_{double} is the number of cells positive for both markers, and the denominator represents the number of cells expressing one marker alone. Lower values of n and s indicate less cross-lineage contamination and thus higher segmentation purity.

Results:
The results are presented in Figure-S6. For the gene pairs Hexb vs. Slc32a1 and Pdgfra vs. Gfap (Panels A and B), we observe that the absolute number of double-positive cells (n) is higher for BOMS, Baysor, and pciSeq compared to the Silver Standard. This increase is expected, as these methods segment a larger number of cells. However, the contamination score (s), which normalizes for the number of cells expressing either marker alone, is slightly lower for BOMS. This indicates that, relative to the increased cell count, BOMS assigns transcripts more accurately, leading to lower cross-lineage contamination. In other words, while more cells are segmented, BOMS ensures that these cells remain transcriptionally coherent.

For the gene pairs Ttr vs. Gad2 and Ttr vs. Pdgfra (Panels C and D), both the absolute number of double-positive cells and the contamination score are lower for BOMS, Baysor, and pciSeq compared to the Silver Standard. These results demonstrate that BOMS more effectively restricts marker co-expression between distinct lineages, underscoring its advantage in producing cleaner, biologically consistent segmentations.

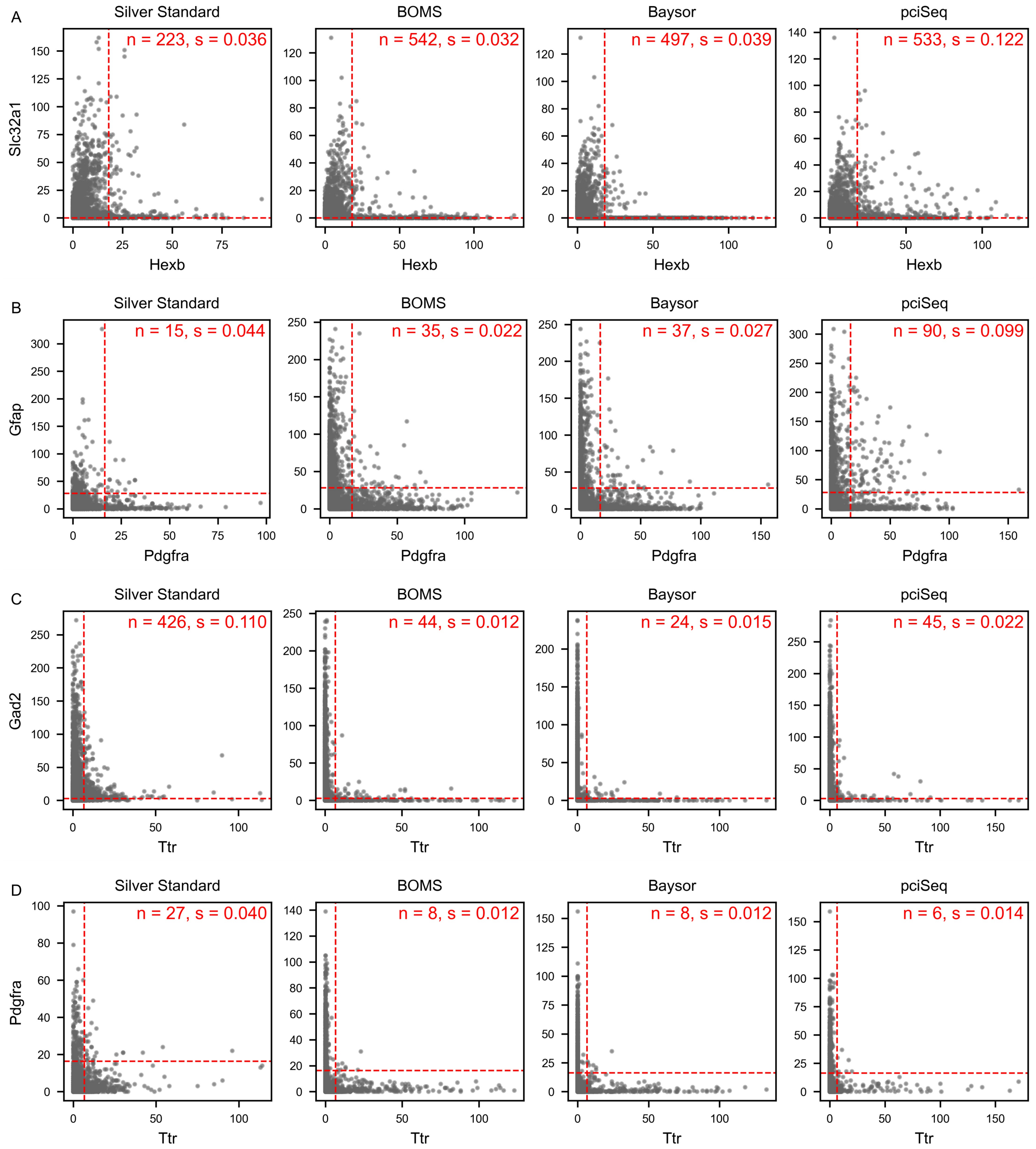

Figure S6. Co-occurrence of marker genes in osmFISH Dataset. Scatter plots showing expression of mutually exclusive lineage markers. Red dashed lines indicate thresholds. Numbers n and scores s represent double-positive cell counts and contamination scores, respectively. A: Hexb (Microglia marker) vs. Slc32a1 (Inhibitory neuronal marker). B: Pdgfra (Oligodendrocyte Precursor cell marker) vs. Gfap (Astrocyte marker). C: Ttr (Choroid plexus epithelial marker) vs. Gad2 (Inhibitory neuronal marker). D: Ttr vs. Pdgfra (Oligodendrocyte Precursor cell marker).
